# Nexus Between Financial Events and Emotional Exhaustion: Mediating Roles of Deliberate Thinking and Negative Interpersonal Events

**DOI:** 10.3389/fpsyg.2022.840701

**Published:** 2022-07-14

**Authors:** Liwei Yang

**Affiliations:** Department of Physical Education, North China Electric Power University, Beijing, China

**Keywords:** emotional exhaustion, financial events, financial stress, deliberate thinking, negative interpersonal events, economic sustainability

## Abstract

Financial stress and emotional exhaustion have become prevalent elements of modern society, especially after COVID-19. This pandemic has changed people’s lives, particularly in a negative way. Individuals have begun to face the stress and emotional exhaustion associated with particular financial stressor events. However, limited studies have analyzed the relationship between financial stressor events and emotional exhaustion to date. Therefore, the current study aims to explore the relationship between different financial stressor events in an individual’s life and emotional exhaustion based on their well-being. This study also identifies the variables that play a mediating role in assessing the relationship between emotional exhaustion and financial stressor events. To achieve this, the researcher collected data from employees working in large organizations in May 2021 and December 2021. The study employs path analysis to assess the relationship between the identified variables. The study found that both organizations and employees are directly affected by financial stress, leading to emotional exhaustion or a decline in the mental well-being of the individuals. In addition, the study also found that financial stress and emotional exhaustion can directly affect the physical health of individuals. The study further revealed that deliberate thinking, negative interpersonal events, and individual characteristics are some of the identified variables that act as mediators between financial stressor events and emotional exhaustion.

## Introduction

Past researchers have established a relationship between financial stress and emotional exhaustion. In support of this statement, further studies have found a clear connection between financial distress/stress caused by financial stressor events and the mental well-being of the individual (Bagwell and Kim, 2003; O’Neill et al., 2006; Prawitz et al., 2006). Recent literature has added the notion that financial stress is a serious condition whereby an individual is pushed to a state of emotional exhaustion. The poor financial state of an individual or a large organization will lead to financial stress which is considered a new phenomenon. It was also found that financial stress is subjective and differs from one person to another. For instance, two individuals earning the same income face different levels of financial stress and emotional exhaustion based on their perceptions regarding their finances.

It is important to note that financial stressor events in an individual’s life mostly last for a short period. However, financial stressor events can sometimes cause enduring effects on individual psychological well-being, leading to emotional exhaustion. Several studies observed that the major cause of financial stress involves several financial stressor events piling up one after another. Negative financial events include receiving due notices from creditors, collection agencies sending overdue notices, falling behind in paying bills or other payments, bouncing checks due to insufficient funds, thinking about family emergencies that would require financial stability, etc.

Primarily, financial stressor events for individuals and organizations in the United States revolve around falling behind in paying credit card bills and meeting other financial obligations. Cases like bankruptcy, credit card debt, insufficient liquid money savings, low savings rate, and low asset accumulation are some of the commonly identified financial stressor events causing financial stress among individuals and organizations at an alarming rate (O’Neill et al., 2006; Prawitz et al., 2006; Abdullah et al., 2021; Bayighomog et al., 2021). At some point in their lives, most adults face issues related to financial stressor events, followed by financial stress. The recent literature stresses the need to understand the relationship between financial stresses due to financial stressor events and its related negative effects on an individual’s mental health. Financial events can lead to the psychological ill health of an individual. The main problem identified is that individuals with excessive financial debt faced more severe health issues than others, which worsened their psychological well-being (Lomas et al., 2018; Ajaz et al., 2020; Shehzad et al., 2020).

To date, little research has been published on understanding the relationship between financial stressor events and the psychological well-being of individuals (Akhavan-Abiri et al., 2019). Although past studies focused on financial stress caused by financial stressor events, there is inadequate knowledge regarding the relationship between financial stressor events and emotional exhaustion and the mediating roles of variables like negative interpersonal events and deliberate thinking. This study identified this research gap, which motivated the researcher to explore this topic in more depth (O’Neill et al., 2005b; Li et al., 2017; Akhavan-Abiri et al., 2019; Loh et al., 2019; Mohsin et al., 2021). Therefore, this study aims to understand the relationship between financial stressor events and the emotional exhaustion of individuals. Furthermore, it also attempts to uncover the variables that explain the relationship between financial stress caused by financial stressor events and emotional exhaustion.

The structure of this manuscript is as follows. Section “Literature Review” of the manuscript provides a literature review of financial stressor events, emotional exhaustion, and the relationship between the two. This section also covers the proposed hypothesis. Section “Methodology” discusses the methodology adopted in the study and the different statistical methods used to analyze the data collected. Section 4 outlines the findings and results of the study. Lastly, section “Conclusion” presents the study’s conclusion along with some relevant recommendations for future research.

## Literature Review

This manuscript begins with a short review of the literature examining the concepts of financial stress, financial stressor events, and the financial behavior of individuals and large organizations. Prior research suggests that well-being is one of the key individual characteristics impacted by stress caused by financial stressor events, in alignment with the financial behavior and perceptions of individuals. The research has also explored how particular financial behaviors relate to an individual’s financial well-being (Bailey et al., 1998; Ghaffar, 2020; Choi and Heo, 2021). Many existing studies have examined the behavior of individuals and organizations regarding the financial aspects of their life and the ways or processes used to obtain success in terms of finance. These studies also examined the multiple ways to manage finances, exploring their direct influence on the psychological well-being of an individual (Portoghese et al., 2017; Sarfraz et al., 2020; Choi and Heo, 2021). There is a wide choice of approaches available in the literature to analyze the role of financial management in the life of individuals, which will eventually define the financial well-being of an individual as an entity (Kim et al., 2003b; O’Neill et al., 2005a). However, there are some common findings concerning the link between an individual’s financial management and their financial well-being and health.

Some of the financial stressor events identified by past research include foreclosing a home, garnishing wages, receiving overdue bills, increasing credit card debt, bankruptcy, insolvency, etc. These financial stressor events cause financial stress to the individual and are commonly identified as non-normative events. It was claimed that financial stressor events lead to financial stress. Hence, the number of financial stressor events reduce the psychological well-being of an individual (Garman and Sorhaindo, 2005; Lyons and Yilmazer, 2005; O’Neill et al., 2005b; Manzano García and Ayala Calvo, 2012). Studies on financial stress are well documented. It is acknowledged that when an individual stresses about financial stressor events, there is a high possibility that this response negatively influences the individual’s body. In addition to this, such individuals gradually develop emotional exhaustion, behavioral issues, physical ailments, and other mind-related injuries (Kim et al., 2003b; Garman and Sorhaindo, 2005; O’Neill et al., 2006). A few other studies also pointed out that even financial counseling agencies and clients face financial stress. Nevertheless, the amount of stress experienced in these scenarios is unidentified.

Financial events such as paying high levels of credit card debt and overdue bills can cause financial stress. The resultant increased levels of financial stress will likely affect mental well-being, leading to emotional exhaustion after a specific amount of time. Therefore, it has been claimed that financial stressor events could negatively influence an individual’s health and functioning (Weisman, 2002; Akhavan-Abiri et al., 2019). Surprisingly, some other studies found that individuals gained relatively higher benefits by receiving counseling on different ways to manage their finances. After receiving the counseling, individuals were found to be able to manage their finances and credit. Sometimes, the creditors reduced the interest rates of the debt, while others eliminated fines. On the other hand, other studies aimed to examine the interrelationship between financial stress, negative financial events, and the psychological well-being of an individual (Bagwell and Kim, 2003; Vlaev and Elliott, 2014; Strömbäck et al., 2017; Lomas et al., 2018; Iramani and Lutfi, 2021).

Most early studies and current work focus on the harmful changes caused to an individual’s health by stress. It is stated that stress expedites growing old by lessening the lifespan of an individual’s cells, leading to the beginning of various diseases (Moon and Hur, 2011; Brüggen et al., 2017). Some other studies identified that stress causes different diseases, such as chest pain, shoulder pain, anxiety, headaches, fatigue, insomnia, nausea, back pain, depression, and so forth (Cropanzano et al., 2003; Halbesleben and Bowler, 2007; Mahendru, 2020). In general, individuals or clients visit a credit counseling agency to learn different ways to manage their debts due to experiencing significant amounts of financial stress. An individual’s substandard financial behaviors negatively affect the well-being of the individual. As a result, they develop a need to find a way to deal with those financial stressor events that cause high levels of stress within the individual. There is very limited literature on the relationship between financial stressor events, financial stress, financial well-being, and the emotional exhaustion of individuals. Issues like bankruptcy, credit card debt, insufficient liquid money savings, low savings rate, and low asset accumulation are commonly cited financial stressor events that result in increased rates of financial stress among individuals and organizations (Mahendru, 2020; Robillard et al., 2020). Most adults encounter challenges related to financial stressor events, followed by financial stress, at some stage of their life. The recent literature highlights the need to understand the relationship between financial stresses caused by financial stressor events and negative effects on an individual’s health.

Several authors have recognized that financial stress causes several health issues (Koesten, 2005; Brüggen et al., 2017). Most importantly, it is noted that financial stress is caused not just solely by an individual’s income, but also depends on the individuals’ ability to cater to their financial responsibilities. Financial stress is affected by other needs, such as physical and psychological ones. As has been previously reported in the literature, inadequate financial well-being produces emotional stress, social stress, and physical stress (Kim et al., 2003a; Vlaev and Elliott, 2014; Brüggen et al., 2017). The financial situation of the individual and their income is commonly used to measure the psychological well-being of that particular individual (Prawitz et al., 2006).

A review of the existing literature shows that the financial well-being of an individual is predominantly interconnected with their psychological well-being. Some promising studies have proven that financial stressor events cause financial stress among individuals, which affects their health in numerous ways (Bailey et al., 1998; Portoghese et al., 2017; Akhavan-Abiri et al., 2019). One such financial stress is identified as credit card debt and the stress associated with that debt. This stress eventually affects the individual’s health (Manzano García and Ayala Calvo, 2012; Ghaffar, 2020). Over time, an extensive body of literature has developed on the levels of financial stress an individual goes through, resulting in physical and mental impairments. Therefore, it is claimed that financial stressor events cause financial stress. Consequently, individuals experiencing them feel emotionally exhausted or burned out. Similarly, a few other studies suggest that financial stress is related to emotional anxiety. As such, it is conceivable that financial stress leads to various mental health issues (Fries and Neal, 2006). One study proposed that continuous telephonic calls from the creditors trying to clear a debt or obtain payment of bills can cause stress within the individual and lead to mental and physical illnesses. Similarly, past research studies have identified that individuals with poor or extremely bad health had high financial stress and vice versa. It was also commented that the most important cause for financial stress is the number of bankruptcy filings (Halbesleben and Bowler, 2007; Vlaev and Elliott, 2014; Mahendru, 2020).

A few other studies have examined stress associated with finance, especially during COVID-19. During the pandemic, it was reported that individuals faced extreme levels of stress. Due to the lockdown measures which caused disruption to income, some individuals became unable to repay loans to financial institutions on time or at all. This caused stress that eventually led to a state of exhaustion. Many individuals found it hard to make a living during this pandemic. Moreover, people deliberately thought of negative aspects which created financial stress and, again, led to emotional exhaustion (Huang and Zhao, 2020; Robillard et al., 2020). Reasons like employment loss, the financial situation, and the psychological distress caused by the pandemic itself sometimes led to severe psychological issues. Overall, it is therefore apparent that financial stressor events cause financial stress, resulting in an individual feeling emotionally exhausted or burned out (Robillard et al., 2020).

**FIGURE 1:**
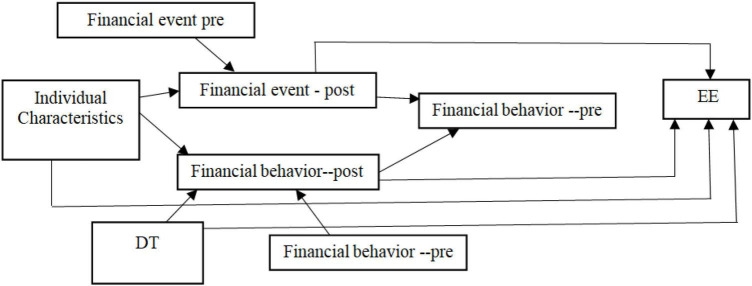
Study research model. EE, Emotional Exhaustion; DT, Deliberate Thinking.

To investigate the relationship between financial stressor events, financial stress, financial well-being, and the emotional exhaustion of employees working in large organizations, the researcher adopts the research model proposed by Kim and Garman (2003). This study examines the relationship between financial stressor events and emotional exhaustion/psychological well-being of the participants and explores the mediating role played by identified variables like deliberate thinking, negative interpersonal events, financial well-being, and financial behavior of the individuals. Figure 1 presents the research model adopted in the current study based on the hypothesis.

Based on the analysis of the literature, the study proposes the following hypothesis:

H1: There is a positive relationship between financial stressor events and the emotional exhaustion of an individual.
H2: There is a positive relationship between financial well-being, financial behavior, and financial stress.
H3: Deliberate thinking plays a mediating role between financial stressor events and emotional exhaustion.
H4: Negative interpersonal events play a mediating role between financial stressor events and emotional exhaustion.

## Methodology

The researcher utilized the knowledge base recorded in the top organizations of the United States that deal with various financial stressor events. Data for the current study was collected two times, with a six-month interval between collections to understand and analyze the trend. In the first data collection procedure, a survey was sent to employees working in large organizations through email in May 2021. To collect the data from 5,000 clients, a sample of 2,000 was finalized. Based on telephone conversations recorded between January 2021 and May 2021, the sample for the current study was determined. The selected sample included individuals and organizations who had agreed to repay their debts to creditors. The mail survey sent across the selected sample included 58 items. From 2,000 surveys, 20% responded to the survey. As mentioned earlier, the second data collection process began 6 months after the first data collection process in December 2021. The second data collection was obtained from the 400 people who responded to the first data collection. Out of 400 people, 60 of them failed to receive the mail as they had changed their address. Out of 340 people who received the second mail, 190 of them returned the surveys. Out of 190 surveys received, the researcher utilized only 173 surveys for data analysis.

The researcher has considered only those participants who responded to the first and second data collection processes. The questionnaire distributed to the selected participants included the unique identification number provided to the individuals, which were eventually used to compare the data collected from both data collections. Out of 173 survey responses received, the active financial debt cases were 70, and 105 of them were inactive by December 2021. The other important point noted by the researcher is that 70 active participants made their payments, while 105 of them did not even pay their first installments. Out of all the active participants, 67% were females and 33% were males, 53% of the respondents are married, while 40% of them are single, and 7% of them are living together with their selected partner. Regarding the age of the respondents, the majority of the respondents belong to the age group of 35 to 40.

Concerning the participant’s educational qualifications, 38% of them completed their diploma, 22.5% completed their graduation, and 24.1% completed their higher education. Of all the active participants, 81% of them either had a full-time job or part-time job, and the annual household income of participants was between $50,000 and $460,000. These are the individual characteristics of the active participants included in the study. The researcher performed two main tests to analyze the data collected, namely, Chi-square and *t*-test. These tests are carried out to identify the difference between the variables identified in the study. There were not many differences in terms of gender, education, marital status, race, age, employment, etc. However, the income of the participants varied the results.

Another important aspect being noted here is that the participant’s characteristics are like that of the total population collected for the study from the organization’s perspective. From the organization’s internal report, it is inferred that 61% of them were females, followed by males; 47% of them were married, followed by singles and living together; and 60% of them completed their graduation or diploma, at least. At the same time, the age of the participants varied between 35 and 40. Of all the participants whose monthly median net income is $2,000, 55% of them had an unsecured debt of $20,000. Majority of the participants had an unsecured debt that constituted 50% of their annual income. Information of those participants who did not respond in the first data collection process is unavailable. Table 1 presents the demographic details of the participants.

**TABLE 1:** Demographics of respondents.

Variable	Number of respondents (%)
**Age (years)**	
18 – 29	0
30 – 39	67.5
40 – 49	22.5
**Gender**	
Male	61
Female	39
Prefer not to say	0
**Education**	
No formal education	0
High school	24.1
College (Bachelor)	22.5
Masters	36
Diploma	38
Others	4
**Income**	
50,000$ and 60,000$	99
Job type	
Part/full time	81
No job	19

The survey questionnaire helped the researcher measure the active participants’ financial behavior. The variables used to measure the financial behavior included the following: “I deliberately think or plan for my financially secured future,” “started thinking to have increased savings as a way to deal with negative events in the life of an individual,” “understood the negative influence of having increased debts and hence reduce them,” “started thinking to have a plan to spend money carefully,” and “deliberately cutting down the daily and living expenses.” Study participants responded to the gender question with “Yes or No”. The items mentioned above were integrated to derive the final financial behavior score, focusing on financial events. In the first data collection process, the financial behavior score of the active participants was 0.74 for deliberate thinking and 0.66 for negative interpersonal events. These variables were measured using Cronbach’s alpha to derive accurate results.

Past studies conceptualized that financial stressor events like bankruptcy, credit card debt, insufficient liquid money savings, low savings rate, low asset accumulation, and so forth have the actual potential to cause financial stress among individuals (Akhavan-Abiri et al., 2019; Bayighomog et al., 2021). Although the degree of financial stressor events and financial stress is not the same, there exists a relationship between them. The active respondents of the present study stated that they had to undergo 22 different types of the financial stress associated with different financial events, such as bankruptcy, credit card debt, insufficient liquid money savings, low savings rate, low asset accumulation, and so forth, in the last year. The financial events recorded had financial stress, which was recorded as 0 and 1, which never meant and once or more than once, respectively. The category of “once” and “more than once” was combined as the study’s primary aim is to find out if there is a more significant relationship between financial stressor events and emotional exhaustion or stress than the degree of each event. The items’ scale, which included 22 items, was adapted from the study of Bagwell (2000) to record the financial stressor score. In the first data collection process (pre), the financial stressor event score of the active participants was 0.81, while it was 0.88 in the second data collection process (post). These variables were measured using Cronbach’s alpha to derive accurate results. The participants stated that the financial stressor events included receiving an overdue notice from the creditors, telephonic calls to pay the due bills, paying due credit card debt, notice for bankruptcy, etc.

The researcher also examined the financial well-being of the participants as these financial events had the potential to cause emotional exhaustion. Financial well-being is understood as the ability to analyze an individual’s financial situation. It is how an individual perceives and understands his or her financial situation. The study measured the financial well-being of the participants by passing the following statements: “I feel stressed and burned out due to my current financial situation,” “I feel I am financially stable and emotionally fit,” “I am feeling bad about my current financial situation,” and “I experience high levels of stress about my personal finance and feel emotionally drained.” The responses to the above questions were recorded as 0 and 1, where 0 represented never and 1 represented once or more than once. From the above responses, the researcher was able to derive the score of financial well-being in relation to financial event and emotional exhaustion. In the first and second data collection process, the financial well-being score of the active participants was recorded as 0.89. These variables were measured using Cronbach’s alpha to derive accurate results. Likewise, the emotional exhaustion and health of the individuals were measured using the following statements: “I started feeling emotionally burned out;” “I started facing various physical and mental illness;” “I am experiencing high levels of stress, which is very rarely found in my age group;” and “Experiencing health problems.” In the first data collection process (pre), the health score of the active participants was 0.66, while it was 0.82 in the second data collection process (post). These variables were measured using Cronbach’s alpha to derive accurate results.

To analyze the data collected in the present study, the researcher employed *t*-test, factor analysis, frequency distribution, correlations, and reliability test for each of the identified variables. All the variables in the study were recorded appropriately. With the intent to find out the differences between financial events, financial well-being, financial stress, financial behavior, and emotional exhaustion in the employees who had financial stress compared to those who did not have, the study employed descriptive analysis and *t*-tests. In addition, the researcher also employed path analysis to measure the research model. SAS System’s CALIS procedure was adapted to analyze the data collected. All the data analysis was performed on the variance and co-variance matrix which utilized probability methods of estimating the parameters.

In this study, the relationships between financial events, financial stress, financial well-being, financial behavior, and emotional exhaustion based on individual characteristics were investigated with the mediating role of deliberate thinking and negative interpersonal events. The significant focus of this study was to analyze the effect of financial stressor events on the emotional well-being of an individual, tracking the mediating role played by different variables, namely, deliberate thinking, negative interpersonal events, financial behavior, individual characteristics, and financial well-being. Interestingly, four regression equations were included in the path analysis. Most importantly, regression coefficients were utilized to describe the path analysis. The dependent variables identified during the first regression analysis were financial behavior and financial well-being, while the dependant variables identified during the second regression analysis were financial events and emotional exhaustion. The dependent variables identified during the third regression analysis were financial stress and deliberate thinking, and the dependent variables identified during the fourth regression analysis were financial stress and negative interpersonal events. On the other hand, the independent variables identified in the study were the individual characteristics that were in line with financial stressor events.

## Findings and Discussion

### Results of *T*-Test and Path Analysis

To examine the relationship between financial events, financial stress, financial well-being, financial behavior, and emotional exhaustion based on the individual characteristics with the mediating role of identified variables like deliberate thinking and negative interpersonal events, the study conducted a *t*-test analysis. Data collected in the first and second data collection processes were included in data analysis. Intending to compare the identified independent and dependent variables scores, the researcher employed a paired *t*-test analysis. This test was conducted for both active and inactive participants and was used to analyze the data collected. Results of the *t*-test analysis demonstrated significant differences in the relationship between the identified variables. Participants actively responding to the financial events stated that they experience less financial stress and high financial well-being. Therefore, they maintained good psychological well-being, unlike inactive participants. It was demonstrated that inactive participants exhibited no major differences or changes in their emotional well-being and financial stressor events. Furthermore, participants who actively respond to the financial events eventually experienced a huge drop in financial stress, thereby allowing them to maintain their emotional well-being.

To assess the mediating roles of deliberate thinking and negative interpersonal events in financial events and examine the relationship between financial events, financial stress, financial well-being, financial behavior, and emotional exhaustion, the researcher employed path analysis. The *T*-test results of the study variables (Financial Behavior, Financial Well-Being, Financial Events, and Emotional Exhaustion) are shown in Table 2. Table 3 presents the *T*-Test results of financial events, deliberate thinking, and negative interpersonal events. Due to missing data, only 137 cases were included.

**TABLE 2:** *T*-test results of financial behavior, financial well-being, financial events, and emotional exhaustion.

	Mean	
	Active group (*n* = 71)	Inactive group (*n* = 102)
**Financial behavior and financial well-being**		
Pre (first data)	3.17	2.78
Post (second data)	3.85	3.23
	*t* = 3.34**	*t* = 2.45*
**Financial events and emotional exhaustion**		
Pre (first data)	9.02	11.01
Post (second data)	5.17	9.76
	*t* = −8.21**	*t* = −3.78**

*p < 0.05, **p < 0.01.

**TABLE 3:** *T*-test results of financial events, deliberate thinking, and negative interpersonal events.

	Mean	
	Active group (*n* = 71)	Inactive group (*n* = 102)
**Financial events and deliberate thinking**		
Pre (first data)	9.58	8.45
Post (second data)	10.78	9.45
	*t* = 2.71*	*t* = 2.41*
**Financial event and negative interpersonal events**		
Pre (first data)	10.62	10.71
Post (second data)	10.92	10.69
	*t* = 2.18*	*t* = −0.38

*p < 0.05, **p < 0.01.

The present study confirmed the findings on the relationship between financial events, financial stress, financial well-being, financial behavior, and emotional exhaustion based on the individual characteristics with the mediating role of identified variables like deliberate thinking and negative interpersonal events. From the first hypothesis, the study identified the dependent variables, such as financial behavior and financial well-being. Independent variables, such as individual characteristics of the participants, exhibited a significant effect on their financial behavior and well-being during the first and second data collection (Mahendru, 2020).

Furthermore, individual characteristics of an individual, such as age, gender, income, and so forth, explicated only 9.1% of the variance in the relationship between financial events and emotional exhaustion. From the results, it can be stated that Hypothesis 1 is true and that there is a positive relationship between financial events and the emotional exhaustion of an individual.

On the other hand, hypothesis 2 identified financial events and emotional exhaustion variables. From the results, it can be inferred that there were no significant changes between the identified variables. However, independent variables, such as the individual characteristics of an individual, exhibited significant changes. It was also found that the financial events that caused stress among the participants tended to exert an influence on individuals, eventually affecting their financial well-being and financial behavior (Akhavan-Abiri et al., 2019; Iramani and Lutfi, 2021). In addition, it was also found that an individual’s financial well-being and financial behavior are related to financial stress, be it directly or indirectly (Bayighomog et al., 2021). Most importantly, the individual characteristics of the participants showed a 28% variance in financial events and hence supports hypothesis 2.

Hypothesis 3 identified variables such as financial stress and deliberate thinking. From the results, it can be inferred that financial stress and deliberate thinking of the participants were interrelated and can be considered as important variables that help the researcher to identify the relationship between financial events and emotional exhaustion. It can also be inferred that deliberate thinking plays a mediating role between financial events and emotional exhaustion. Participants who exhibited deliberate thinking had higher levels of financial stress, thereby affecting their financial well-being. Likewise, participants who had positive financial behavior experienced more positive financial well-being than others. It was also found that deliberate thinking has a direct impact on the financial well-being of the individual and an indirect impact on the financial events. Most importantly, the individual characteristics of the participants showed 32.3% of variance in financial events with the mediating role of deliberate thinking, thereby partially supporting hypothesis 3.

**FIGURE 2:**
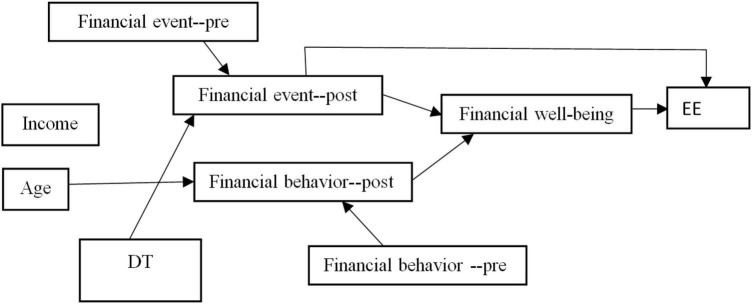
Path analysis. EE, Emotional Exhaustion; DT, Deliberate Thinking.

Hypothesis 4 identified variables such as financial stress and negative interpersonal events. From the results, it can be inferred that financial stress and negative interpersonal events were interrelated and can be considered as important variables that help the researcher identify the relationship between financial events and emotional exhaustion. It can also be inferred that negative interpersonal event plays a mediating role between financial events and emotional exhaustion. Participants who exhibited negative interpersonal events had higher levels of financial stress, which affects their financial well-being (Lomas et al., 2018). Likewise, participants who had positive financial behavior experienced a better financial well-being than others. It was also found that negative interpersonal events have a direct impact on the financial well-being of the individual and an indirect impact on the financial events. Most importantly, the individual characteristics of the participants showed 29.1% of variance in financial events with the mediating role of deliberate thinking, thereby partially supporting hypothesis 4. Table 4 presents the path analysis results of financial behavior, financial well-being, financial events, and emotional exhaustion, while Table 5 shows the path analysis results of financial events, deliberate thinking, and negative interpersonal events. Figure 2 shows the graphical representation of path analysis test.

**TABLE 4:** Path analysis results of financial behavior, financial well-being, financial events, and emotional exhaustion.

	Income	Age	FS–pre	FB–pre	CC	FS–post	FB–post	FW	*R2*
Financial behaviors and financial well-being	0.043	0.172*	–	0.140**	0.091	–	–	–	0.112
Financial stressor events and Emotional Exhaustion	−0.098	−0.145	0.401***	–	−0.245**	–	–	–	0.298

*p < 0.05, **p < 0.01, and ***p < 0.001.

**TABLE 5:** Path analysis results of financial events, deliberate thinking and negative interpersonal events.

	Income	Age	FS–pre	FB–pre	CC	FS–post	FB–post	FW	*R2*
Financial events and deliberate thinking	−0.041	0.131	–	–	−0.098	0.491***	0.287***	–	0.341
Financial event and negative interpersonal events	0.081	−0.051	–	–	0.002**	−0.198*	0.091	0.387***	0.281

*p < 0.05, **p < 0.01, and ***p < 0.001.

Direct and Indirect effects of financial stressor events impact the participant’s emotional well-being. In other words, there defiantly exists a relationship between individual’s financial stressor events and emotional exhaustion which results in various psychological and physical related problems. The results of the *t*-test proved that there were significant differences in the relationship between the identified variables. Participants who actively responded to the financial events stated that they experienced less financial stress, high financial well-being, and, therefore, maintained good psychological well-being unlike inactive participants. It was demonstrated that inactive participants exhibited no major differences or changes about their emotional well-being and financial stressor events. Furthermore, participants who actively responded to the financial events eventually experienced a huge drop in financial stress, thereby allowing them to maintain their emotional well-being. Other findings of the *t*-test analysis included how active participants had better psychological well-being than those who were inactive for various reasons. Hence, financial stressor events are interrelated with financial well-being, financial stress, financial behavior, and emotional exhaustion of the individual or organization. While the results of path analysis proved that hypotheses H1 and H2 are accepted, hypotheses H3 and H4 are partially accepted. Although the results of this study are not consistent with that of the past research, it remains true as per the current study’s findings.

## Conclusion

The manuscript concludes by arguing that there is a positive relationship between financial stressor events, financial stress, financial well-being, financial behavior, and emotional exhaustion based on an individual’s characteristics, with the mediating role of identified variables like deliberate thinking and negative interpersonal events. From the study, it can be concluded that deliberate thinking and negative interpersonal events act as mediators between financial stressor events and the emotional exhaustion of an individual. However, these results cannot be generalized as individual characteristics vary from one person to another, causing significant adjustments to the proposed hypothesis. The results indicate that the relationship between financial stressor events and the emotional exhaustion of an individual can be affected by various factors and not just the variables of deliberate thinking and negative interpersonal events.

## Data Availability Statement

The raw data supporting the conclusions of this article will be made available by the authors, without undue reservation.

## Ethics Statement

Ethical review and approval was not required for the study on human participants in accordance with the local legislation and institutional requirements. The patients/participants provided their written informed consent to participate in this study.

## Author Contributions

The author confirms being the sole contributor of this work and has approved it for publication.

## Conflict of Interest

The author declares that the research was conducted in the absence of any commercial or financial relationships that could be construed as a potential conflict of interest.

## Publisher’s Note

All claims expressed in this article are solely those of the authors and do not necessarily represent those of their affiliated organizations, or those of the publisher, the editors and the reviewers. Any product that may be evaluated in this article, or claim that may be made by its manufacturer, is not guaranteed or endorsed by the publisher.
